# Convolutional neural networks in paediatric fracture detection: pooled evidence from a systematic review and meta-analysis

**DOI:** 10.1007/s00330-026-12462-2

**Published:** 2026-03-21

**Authors:** Alina Pervez, S. Umar Hasan, Alan R. Norrish

**Affiliations:** 1 Department of Trauma and Orthopaedics, Queen’s Medical Centre, Nottingham University Hospitals NHS Trust, Nottingham, United Kingdom; 2 NIHR Biomedical Research Unit (MSK Theme), Academic Unit of Injury, Recovery and Inflammation Sciences, School of Medicine, Faculty of Medicine and Health Sciences, University of Nottingham, Queen’s Medical Centre, Nottingham, United Kingdom

**Keywords:** Fractures, Paediatrics, Radiography, Artificial intelligence, Neural networks

## Abstract

**Objective:**

The objective of this review was to systematically evaluate the diagnostic accuracy of artificial intelligence (AI) models for detecting paediatric appendicular fractures on plain radiographs.

**Materials and methods:**

This review followed the PRISMA-DTA guidelines. MEDLINE, Scopus, Cochrane Library, and Web of Science were searched from inception to May 2025. Eligible studies included paediatric patients (< 21 years) where AI models assessed plain radiographs for fractures, using human readers as the reference standard. Primary outcomes were pooled sensitivity, specificity, diagnostic odds ratio (DOR), positive likelihood ratio (LR^+^), and negative likelihood ratio (LR⁻). The risk of bias was assessed using QUADAS-2. Random-effects models and hierarchical summary receiver operating characteristic (HSROC) curves were applied.

**Results:**

Seventeen studies met the inclusion criteria, with 11 contributing to the meta-analysis (over 10,000 radiographs). Pooled sensitivity was 0.92 (95% CI: 0.89–0.94), and specificity was 0.90 (95% CI: 0.85–0.94), corresponding to a false-positive rate of 0.10. The HSROC curve demonstrated high overall discriminative ability. Subgroup analyses showed comparable diagnostic performance for upper extremity fractures (sensitivity 0.91, specificity 0.89) and lower extremity fractures (sensitivity 0.89, specificity 0.94). The pooled DOR was 104.6, LR^+^ was 9.32, and LR⁻ was 0.089. Most studies had a low risk of bias, though many were retrospective and single-centre with limited external validation.

**Conclusion:**

AI models, particularly deep learning architectures, demonstrate high diagnostic accuracy for detecting paediatric appendicular fractures on radiographs, approaching expert-level performance and improving the diagnostic abilities of junior clinicians. However, broader clinical adoption requires robust external validation and prospective integration into clinical workflows.

**Key Points:**

***Question*** What is the diagnostic accuracy of artificial intelligence models for detecting paediatric appendicular fractures on plain radiographs?

***Findings*** AI models showed high diagnostic accuracy for paediatric appendicular fractures, with a pooled sensitivity of 0.92, specificity of 0.90, strong HSROC performance, and consistent results across limb subgroups.

***Clinical relevance*** AI-assisted fracture detection may improve diagnostic accuracy, support junior clinicians, and reduce delays in identifying paediatric appendicular fractures, enhancing patient safety and enabling faster, more efficient care pathways in emergency and outpatient settings.

## Introduction

Fractures are among the most common childhood injuries, with studies showing that up to 60% of boys and 40% of girls experience at least one before adulthood [1]. The appendicular skeleton is most frequently affected, with distal radius fractures alone making up around a quarter of cases [2]. These injuries are clinically significant and a major cause of long-term morbidity, making timely diagnosis crucial to avoid complications [3, 4].

Plain radiography is the first-line imaging tool for suspected fractures, but interpreting paediatric X-rays can be challenging, especially in busy emergency departments (ED). Several factors contribute to diagnostic difficulty, including suboptimal patient positioning, the absence of comparison films (due to efforts to minimise radiation), delay to reporting leading to missed fractures, and the complex, evolving anatomy of growing bones [5]. Subtle fractures like buckle or growth plate injuries are often missed, particularly by non-specialists, with studies reporting up to 10% of fractures being overlooked on initial ED review [6–9]. This diagnostic gap underscores the need for strategies to augment fracture detection in the paediatric ED setting.

In recent years, artificial intelligence (AI), particularly deep learning with convolutional neural networks (CNNs), has shown promise in enhancing diagnostic accuracy in medical imaging. AI models have demonstrated performance comparable to radiologists in adult fracture detection, with sensitivities and specificities nearing 96% and 94%, respectively [10, 11]. Recent studies show that modern AI models can match or exceed the performance of less-experienced clinicians in detecting limb fractures on X-rays [12–14]. While most AI tools have focused on adult populations [15], interest in paediatric applications is growing. Early results are promising; one deep learning model achieved over 90% sensitivity and specificity for paediatric fractures [15], and another study showed AI as a second reader improved an emergency physician’s sensitivity and overall accuracy [10]. These findings suggest AI could help reduce missed fractures, boost diagnostic confidence, and streamline paediatric fracture workflows.

However, most research to date focuses on adults, and evidence on AI performance for paediatric fractures remains limited. A 2022 review identified only nine paediatric studies and highlighted concerns regarding heterogeneity, limited external validation, and uncertain generalisability of fracture-detection models [16]. To better characterise this gap, we conducted a systematic review and meta-analysis to synthesise the diagnostic accuracy of AI models for paediatric appendicular fracture detection using a methodologically consistent per-radiograph framework, while characterising limitations related to external validation and generalisability.

## Materials and methods

### Eligibility criteria

This review followed PRISMA-DTA guidelines [17], using the PICOS framework for study selection. Population (P): Paediatric patients (< 21 years), with the upper age limit chosen to account for late skeletal maturity, particularly the medial clavicular physis, which fuses around age 21 [18]. Intervention (I): The index test was the use of AI models assessing plain radiographs for fractures. Comparator (C): Human graders as the reference standard. Outcomes (O): Diagnostic accuracy measures, including sensitivity, specificity, accuracy, area under the curve (AUC), true positive (TP), false positive (FP), false negative (FN), and true negative (TN) per radiograph. Studies with quantitative data were meta-analysed; others were included in the qualitative review. Study design (S): RCTs, cohort, and observational studies in English with no time restrictions.

Exclusion criteria were as follows: Studies involving adults, or mixed populations (paediatric and adult), where paediatric data could not be separately extracted. Studies using an index test other than an AI model or a reference standard other than a human grader. Studies where diagnostic accuracy was not a primary or secondary outcome. Review articles, case reports, and case series. Studies evaluating surrogate outcomes (e.g., effusion detection), fracture healing prediction, or multi-class classification without a binary fracture presence/absence endpoint were excluded from quantitative synthesis and discussed qualitatively only.

### Outcomes

The primary outcome was the diagnostic accuracy of AI models in detecting paediatric appendicular fractures per radiograph, measured by sensitivity, specificity, diagnostic odds ratio (DOR), and likelihood ratios (LR^+^ and LR^−^). Secondary outcomes included region-specific performance by anatomical site (e.g., wrist, forearm, ankle). When confusion matrix data (TP, FP, FN, TN) were unavailable, sensitivity and specificity were used to reconstruct it for analysis.

### Search strategy

A comprehensive search was conducted on May 5, 2025, by two authors across MEDLINE, Scopus, Cochrane Library, and Web of Science, from inception to date. Boolean operators were used to combine terms related to “fracture”, “artificial intelligence”, “imaging”, and “children”, following Shelmerdine et al [16]. Reference lists of included studies were also screened for additional eligible articles. The search strategy was designed to capture peer-reviewed diagnostic accuracy studies evaluating AI-based fracture detection and did not include regulatory databases, device registries, or unpublished commercial reports, which are outside the scope of PRISMA-DTA–guided systematic reviews.

### Data extraction

Data were extracted using a standardised form and included key study characteristics (aim, setting, country, inclusion/exclusion criteria), participant demographics (sample size, age, gender distribution), fracture and imaging details (fracture site, number of radiographs), AI model information (model type, dataset origin, training/validation/testing details), diagnostic accuracy metrics (sensitivity, specificity, accuracy, AUC, PPV, NPV, TP, FP, FN, TN), and data required for risk of bias assessment.

### Statistical analyses

The primary diagnostic accuracy data analysed were TP, TN, FP, and FN. DORs were computed and presented using forest plots. The analysis performed was fracture detection per radiograph instead of fracture detection per patient. A bivariate analysis of sensitivity and specificity was performed using maximum likelihood estimation to construct HSROC curves. This model was run with 5000 iterations, a burn-in of 1000, and three chains. Summary measures such as LR^+^, LR^−^, and DOR were calculated using a random-effects model via the DerSimonian–Laird method, with 95% confidence intervals and a continuity correction of 0.5 applied where necessary. Subgroup analyses were performed by anatomical region (upper vs. lower limb). Assessment of small-study effects using Deeks’ funnel plot asymmetry test was not performed due to the limited number of included studies. All analyses were conducted using R version 4.x with RStudio (Version 1.4.1717) as the integrated development environment. Similar methods and statistical analysis have also been previously used by other studies [19, 20].

### Methodological quality

The methodological quality of the included studies was assessed by two users using the Quality Assessment of Diagnostic Accuracy Studies-2 (QUADAS-2) tool by two independent reviewers [21]. This tool evaluates four domains: “patient selection”, “index test”, “reference standard”, and “flow and timing”. Each domain was assessed for risk of bias and rated as “low”, “high”, or “unclear”. Additionally, the first three domains were evaluated for concerns regarding applicability. Discrepancies between reviewers were resolved through consensus.

## Results

### Study selection

**Fig. 1:**
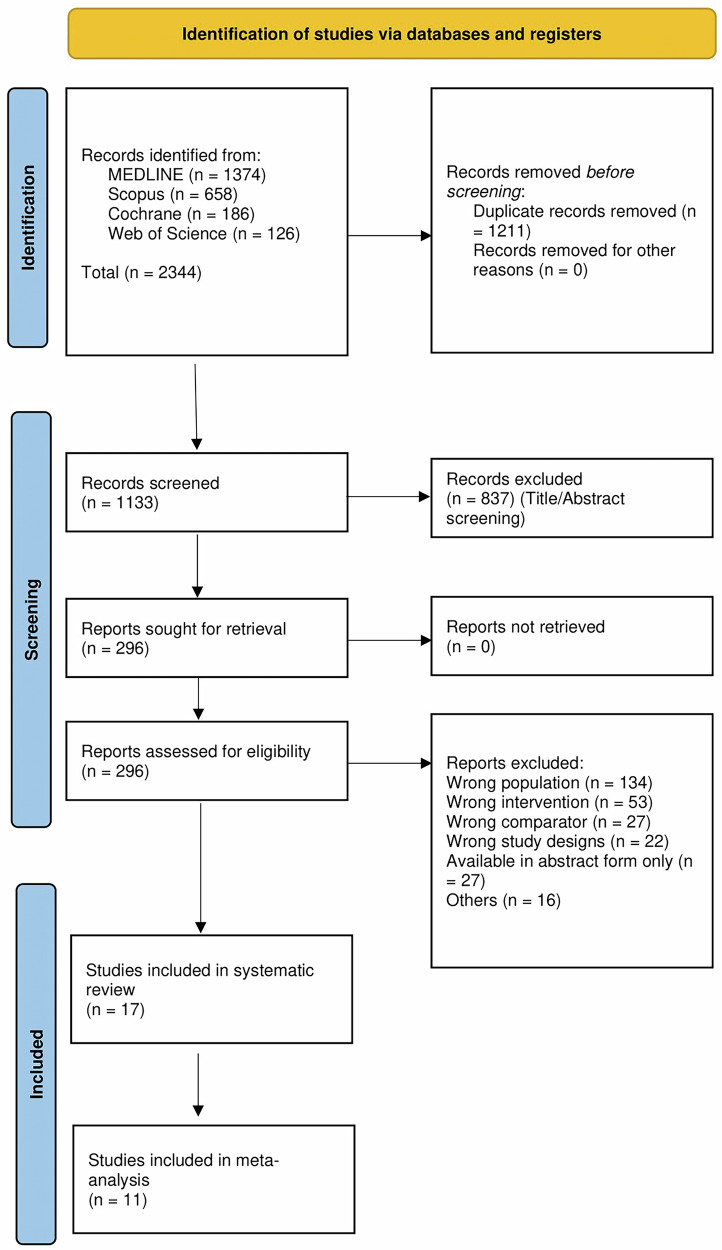
PRISMA 2020 flow diagram illustrating the selection process of studies included in the systematic review and meta-analysis. A total of 2344 records were identified through four databases, of which 1133 were screened and 17 met the inclusion criteria. Eleven studies provided sufficient quantitative data for meta-analysis

The literature search, conducted on 5th May 2025 using the aforementioned strategy, initially retrieved 2344 articles. After removing 1211 duplicates, 1133 unique records remained for screening. Following title and abstract screening, 837 articles were excluded based on relevance, leaving 296 articles for full-text review. Of these, 17 studies met the eligibility criteria and were included in the systematic review [10, 13, 15, 22–35]. Among them, 11 studies provided sufficient quantitative data to be included in the meta-analysis [10, 13, 27–35]. The study selection process is illustrated in Fig. 1 (PRISMA flow diagram).

### Demographic data

Most datasets originated from the U.S. (*n* = 8) [15, 23, 26, 31–35], followed by Austria (*n* = 3) [22, 24, 27]. Patients ranged from 0 to 21 years, with a male majority. Most studies were conducted at single-centre tertiary paediatric hospitals. Radiologists served as the ground truth in all but two studies; in the remaining two studies, orthopaedic surgeons served as the ground truth. In total, 16,514 images were used for testing (The reported total of 16,514 test images refers only to studies included in the quantitative meta-analysis and does not represent the aggregate across all studies in the systematic review). Most studies included training, validation, and testing phases (Tables 1 and 2).

**Table 1:** Demographics

Author, year	Dataset from country	Age range (years)	Male percentage (%)	Number of centres	Types of centres	Body part imaged	Ground truth
Zhou, 2016	USA	1–18	NA	Single	Tertiary Paediatric	Forearm	Radiologist
Malek, 2016	Malaysia	0–12	NA	Single	Tertiary Paediatric	Lower limb	Orthopaedic surgeon
England, 2018	USA	1–9	64.6	Single	Tertiary Paediatric	Elbow	Radiologist
Rayan, 2019	USA	0–18	57	Single	Tertiary Paediatric	Elbow	Radiologist
Choi, 2020	South Korea	0–19	NA	Two centres, same city	Tertiary Paediatric	Elbow	Radiologist
Starosolski, 2020	USA	6.4 (4.4)	33%	Single	Tertiary Paediatric	Distal tibia	Radiologist
Dupuis, 2022	France	0–17	57.3	Single	Tertiary Paediatric	Appendicular skeleton	Radiologist
Tsai, 2022	USA	0.2–11.6	71.3	Single	Tertiary Paediatric	Distal tibia	Radiologist
Zech, 2023 (a)	USA	0.8–17.8	63	Single	Tertiary Paediatric	Wrist	Radiologist
Zech, 2023 (b)	USA	0–21	59.7	Single	Tertiary Paediatric	Upper limb	Radiologist
Chen, 2024	Bangladesh and Austria	NA	NA	Two datasets	Tertiary Paediatric	Appendicular skeleton	Radiologist
Gasmi, 2023	France	< 18	54	Single	Tertiary Paediatric	Appendicular skeleton	Radiologist
Ju, 2023	Austria	0.2–19	56	Single	Tertiary Paediatric	Wrist	Radiologist
Kavak, 2024	Turkey	2–18	NA	Single	Tertiary Paediatric	Appendicular skeleton	Radiologist
Binh, 2024	Austria	0.8–18.9	63.2	Single	Tertiary Paediatric	Distal forearm	Orthopaedic surgeon
Hayashi, 2022	USA	2–21	55.7	Single	Tertiary Paediatric	Appendicular skeleton	Radiologist
Franco, 2024	Italy	0–17	52	Single	Tertiary Paediatric	Appendicular skeleton	Radiologist

*NA* not applicable

**Table 2:** Dataset used and AI training details

Author, year	Imaging used	Total dataset images	Test dataset images	Test type	Unit of analysis	Primary task
Zhou, 2016	Plain radiograph	226	NA	Internal	Per radiograph	Fracture subtype
Malek, 2016	Plain radiograph	57	NA	Internal	Per Patient	Healing time classification
England, 2018	Plain radiograph	901	NA	Internal	Per radiograph	Surrogate
Rayan, 2019	Plain radiograph	58,817	1106	Internal	Per radiograph	Binary fracture detection
Choi, 2020	Plain radiograph	1619	258	External	Per radiograph	Binary fracture detection
Starosolski, 2020	Plain radiograph	2218	98	Internal	Per radiograph	Binary fracture detection
Dupuis, 2022	Plain radiograph	5865	5865	External	Per radiograph	Binary fracture detection
Tsai, 2022	Plain radiograph	250	250	Internal	Per radiograph	Binary fracture detection
Zech, 2023 (a)	Plain radiograph	395	125	Internal	Per radiograph	Binary fracture detection
Zech, 2023 (b)	Plain radiograph	58,846	1334	Internal + External	Per radiograph	Binary fracture detection
Chen, 2024	Plain radiograph	20,327	NA	Public dataset benchmarking only	Per radiograph	Object detection
Gasmi, 2023	Plain radiograph	1310	1310	External	Per radiograph	Binary fracture detection
Ju, 2023	Plain radiograph	20,327	NA	Internal	Per examination	Object detection
Kavak, 2024	Plain radiograph	5150	5150	Internal	Per radiograph	Binary fracture detection
Binh, 2024	Plain radiograph	7006	88	Internal	Per radiograph	Binary fracture detection
Hayashi, 2022	Plain radiograph	300	300	External	Per examination	Binary fracture detection
Franco, 2024	Plain radiograph	1684	600	External	Per radiograph	Binary fracture detection

*NA* not available

### Qualitative analysis

Several studies undertook large-scale, retrospective validations of commercial AI tools like Rayvolve® and BoneView™ [15, 28, 30], while others developed in-house models using institution-specific datasets [31, 32]. This combination of external validation and custom development highlights the need for both reliable clinical tools and continued innovation. Most studies used CNNs, though architectures varied; from Faster R-CNN [35] and YOLOv8 [10] to hybrid CNN–RNN combinations [31] mimicking expert multi-view analysis. AI performance often matched that of junior clinicians but generally lagged behind experienced paediatric specialists.

In terms of anatomical focus, the studies primarily addressed appendicular skeleton injuries, with particular emphasis on the wrist, forearm, elbow, ankle, and shoulder. Some studies tackled diagnostically challenging cases like buckle fractures [34] or classic metaphyseal lesions [33], underscoring AI’s potential utility not only in routine fracture triage but also in identifying subtle and clinically significant pathologies. Diagnostic accuracy was highest for well-defined fractures, with slightly lower performance for subtle or age-specific patterns such as avulsion injuries.

### Quantitative analysis

#### Overall diagnostic accuracy

**Fig. 2:**
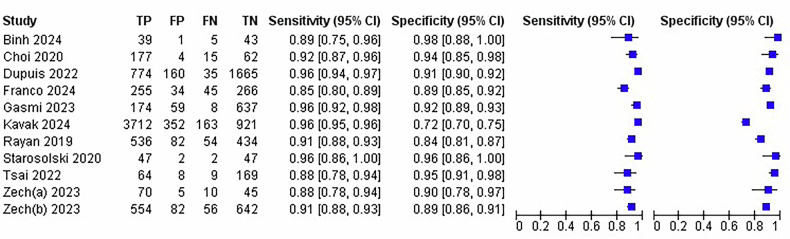
Forest plot of sensitivity and specificity for AI-based detection of paediatric appendicular fractures across 11 included studies. Each square represents a point estimate for a study, and horizontal lines denote the 95% confidence intervals

**Fig. 3:**
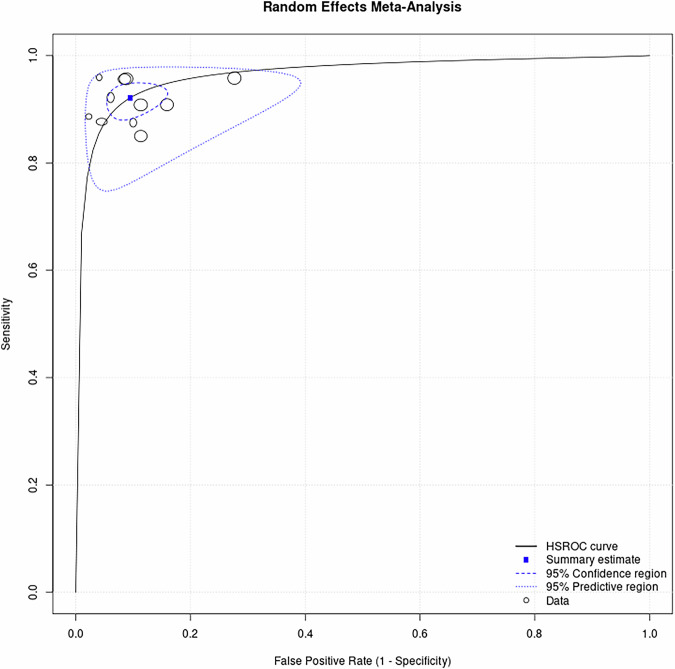
HSROC curve showing pooled sensitivity and specificity of all included studies. The confidence region (solid line) and prediction ellipse (dashed line) reflect precision and between-study variability

Eleven studies [10, 13, 27–35] encompassing more than 10,000 paediatric radiographs assessed for appendicular fractures were included in the meta-analysis. Pooled sensitivity was 0.92 (95% CI: 0.89–0.94), and specificity was 0.90 (95% CI: 0.85–0.94), with a false-positive rate of 0.098 (95% CI: 0.06–0.15) (Fig. 2). The hierarchical summary receiver operating characteristic (HSROC) curve (Fig. 3) showed high overall diagnostic accuracy, with measurable between-study variability, as evidenced by tight confidence regions and clustering in the upper-left ROC quadrant. The bivariate HSROC model demonstrated non-zero between-study variance for both logit sensitivity and specificity, with a moderate correlation between them, indicating residual heterogeneity across studies. The correlation between logit sensitivity and specificity and visual inspection of the HSROC suggested possible threshold effects across studies. Additional metrics: LR^+^ of 9.32 (SD 2.22), LR^−^ of 0.089 (SD 0.016), and the diagnostic odds ratio (DOR) of 104.6 (SD 31.3) underscore the strong diagnostic performance of AI models.

#### Upper limb

**Fig. 4:**
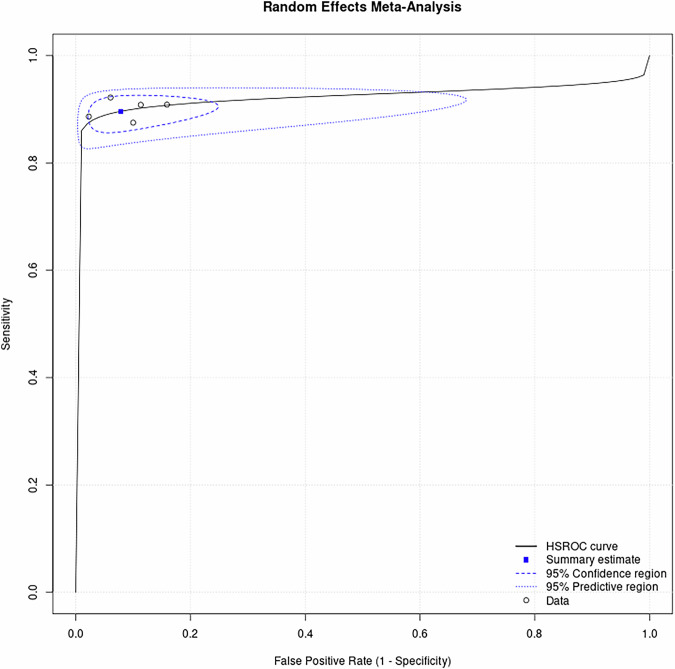
HSROC curve for upper extremity fracture detection. The plot shows high clustering of studies in the upper-left quadrant, indicating strong diagnostic performance of AI models in identifying upper limb fractures

A subgroup meta-analysis of five studies [13, 27, 31, 34, 35] on upper extremity fractures (wrist, forearm, elbow, shoulder) included over 2500 paediatric radiographs. Pooled sensitivity was 0.91 (95% CI: 0.87–0.94) and specificity was 0.89 (95% CI: 0.82–0.94). The estimated false-positive rate was 0.1 (95% CI: 0.06–0.12), indicating a high capacity for correctly ruling out non-fracture cases. The HSROC curve (Fig. 4) showed strong diagnostic performance, with tight clustering across different AI models and datasets. Diagnostic strength was further supported by an LR^+^ of 8.6 (SD 2.7), LR^−^ of 0.10 (SD 0.02), and DOR of 83.2 (SD 32.1), indicating high reproducibility and accuracy of AI in detecting upper extremity fractures in children.

#### Lower limb

**Fig. 5:**
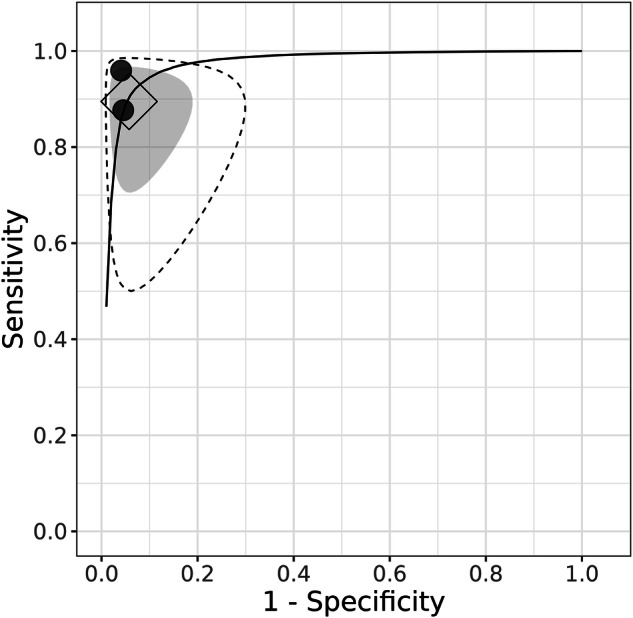
HSROC curve for lower extremity fracture detection. Due to the limited number of included studies (*n* = 2), prediction regions could not be reliably estimated, and only confidence regions are shown

This subgroup analysis included two studies [32, 33] with more than 300 paediatric radiographs. The pooled sensitivity was 0.89 (95% CI: 0.64–0.96), and the pooled specificity was 0.94 (95% CI: 0.70–0.98), indicating that AI models demonstrated robust performance in accurately detecting lower limb fractures with a low false-positive rate of 0.06 (95% CI: 0.02–0.3). The HSROC curve (Fig. 5) visually supports these findings. Prediction regions could not be reliably estimated for the lower limb subgroup due to the limited number of included studies (*n* = 2) and resulting model non-convergence. The corresponding diagnostic performance measures included an LR^+^ of 15.1 (SD 9.8), an LR^−^ of 0.11 (SD 0.09), and a diagnostic odds ratio (DOR) of 132.9 (SD 162.7).

### Methodological quality

**Fig. 6:**
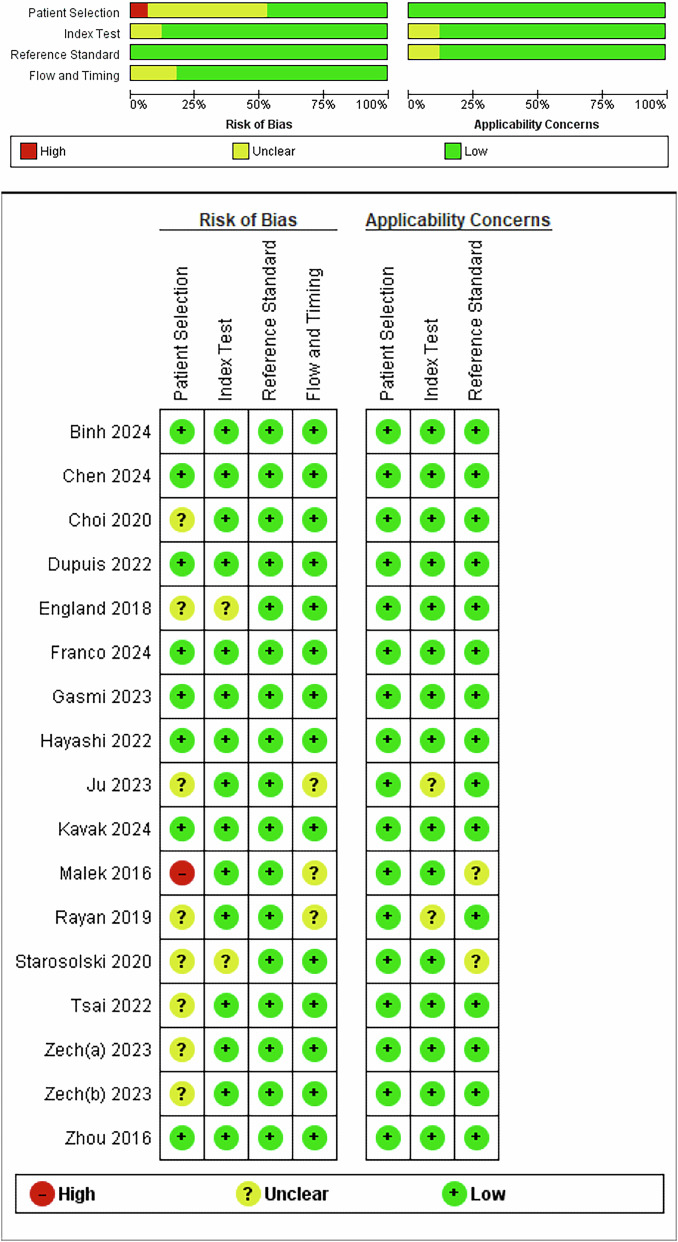
Risk of bias and applicability concerns summary across all included studies, assessed using the QUADAS-2 tool. Most studies were rated low risk in all domains, with minor concerns identified for patient selection and reference standards. Each domain is assessed for risk of bias (left) and applicability (right). Green indicates low risk/concern, yellow indicates unclear, and red indicates high risk

The overall methodological quality was high, as assessed by the QUADAS-2. Most studies had a low risk of bias across all four domains. Patient selection was low risk in 83% of studies, with one (Malek 2016) rated high risk due to unclear recruitment. The index test domain was low risk in all but one study, with some unclear ratings due to insufficient detail on blinding. Most studies also showed low risk for the reference standard, though one had high risk due to inconsistent application. Flow and timing were generally low risk, with a few cases of unclear timing. Applicability concerns were low across studies, with only minor uncertainties due to limited reporting. Full assessments are shown in Fig. 6.

## Discussion

This review demonstrates that AI models can detect paediatric appendicular fractures on radiographs with high diagnostic accuracy, nearing human experts. The primary contribution of this study lies in reducing unit-of-analysis bias through strict adherence to PRISMA-DTA guidance, while transparently highlighting the persistent lack of external validation and its implications for generalisability. Our pooled sensitivity (~ 92%) and specificity (~ 90%) indicate that modern deep learning algorithms correctly identify the vast majority of fractures while maintaining a low false-positive rate. This level of performance is comparable to what has been reported for experienced radiologists and orthopaedic clinicians in fracture detection [36]. In fact, prior studies in adults have shown that several AI systems have performed on par with or even outperformed non-specialist physicians in identifying fractures [37–39]. Consistent with those findings, we observed that current paediatric-focused AI algorithms generally achieve at least similar accuracy to general physicians or junior doctors. Notably, in the limited studies that have directly compared AI systems with subspecialist paediatric radiologists, no statistically significant advantage for AI has been demonstrated to date [16]. This underscores that while some AI models may achieve expert-level performance, there is currently no evidence that they consistently surpass subspecialist readers.

Recent reviews on this topic further highlight the importance of methodological rigour. Two recently published articles by Ashworth et al [40] and Ximenes et al [41] evaluated AI for paediatric fracture detection but did not follow PRISMA-DTA guidelines; instead, they applied the standard PRISMA framework despite focusing on diagnostic accuracy studies. PRISMA-DTA explicitly recommends that reviewers report whether diagnostic accuracy is measured on a per-patient or per-radiograph basis, and warns that pooling across these units can introduce bias (items 13 in Tables 1 and 2 of PRISMA-DTA guidelines) [17]. These studies failed to weigh in the importance of clustering and generalisability bias by including both per-patient and per-radiography studies in the meta-analysis and not forming a stringent eligibility criterion. Pooling per-patient with per-radiograph data risks unit-of-analysis error, inflated precision, and loss of clinical meaning, producing pooled estimates that look more reliable than they really are [42].

For example, Ximenes et al [41] included 16 studies in their meta-analysis, six [15, 43–47] of which reported per-patient measures, while the remaining 10 reported per-radiograph measures. However, the authors did not specify which unit of analysis they prioritised and did not address this issue in their methods or discussion. Similarly, Ashworth et al [40], while conducting a systematic review without meta-analysis, did not account for this problem when formulating eligibility criteria. As a result, the findings of these reviews may be affected by clustering bias, over-estimation of precision, and limited generalisability. By contrast, our review applied PRISMA-DTA guidance strictly, set a clear eligibility framework, and analysed studies according to their unit of assessment, thereby reducing the risk of such biases.

AI’s value is especially clear in supporting less-experienced readers. Multiple studies in our review showed improved diagnostic sensitivity when clinicians used AI as a second reader [10, 34]. For example, Kavak et al [10] reported that AI assistance raised an emergency physician’s sensitivity from 93.7% to 97.0%, and overall accuracy from 88.0% to 94.9%. Such findings highlight AI’s potential as a diagnostic “safety net” in paediatric emergency settings, helping to bridge the gap in expertise between generalists and paediatric radiology specialists. The data suggests that AI is capable of bringing junior or non-specialist performance closer to expert levels, rather than a stand-alone replacement for expert clinical judgement.

Despite promising diagnostic performance, significant limitations remain in the datasets used to train and validate these AI models. Most algorithms were developed on narrow, single-institution datasets with little external validation, raising concerns about overfitting and limited generalisability. Only a few studies used open-access or multicentre data [40], which limits the ability of these models to perform reliably across diverse populations, imaging protocols, or equipment. This lack of diversity may lead to models that perform well internally but struggle with different populations, imaging protocols, or equipment. Prior work has shown that AI accuracy often drops when tested on external datasets [48]. As highlighted by Shelmerdine et al (2022) [16], the heterogeneity of studies and lack of external validation make it difficult to assess generalisability. Our findings echo this—AI models show promise, but their real-world robustness remains unproven. Rigorous external validation using multicentre cohorts is essential before clinical deployment [16].

Beyond accuracy, integrating AI into paediatric fracture diagnosis raises important ethical and practical concerns. A key issue is trust—clinicians must have confidence in AI without over-relying on it. Overdependence can lead to missed fractures or unnecessary treatment, and studies show that inaccurate AI outputs may mislead clinicians [49, 50]. Clear clinical protocols should define how AI findings are to be used—for example, as an aid that must be corroborated by a physician, rather than an arbiter of truth. Closely related is the question of responsibility: if an AI misses a fracture that a radiologist also overlooks because they trusted the AI, who is liable for the error? At present, responsibility remains with the clinician, raising medico-legal challenges that require formal guidance. Ethical use also demands transparency and bias mitigation. Since paediatric populations are diverse, models trained on narrow datasets may underperform in underrepresented groups. Ensuring representative data and regular bias checks is essential for equity. Ultimately, successful AI implementation requires not only strong performance but also clinician training, explainable systems, and robust oversight to build appropriate trust.

Our study has several key strengths. We employed a broad search strategy and included studies up through 2024, resulting in a substantially larger and more accurate evidence base compared to Shelmerdine et al [16], Ashworth et al [40], and Ximenes et al [41]. By adhering to PRISMA-DTA guidelines and performing a thorough QUADAS-2 assessment of methodological quality, we ensured a rigorous and transparent approach to study selection, data extraction, and bias evaluation compared to recent review articles. Furthermore, we conducted detailed subgroup analyses and sensitivity analyses to explore heterogeneity. These methods allowed us to identify performance differences by anatomical region and to explore potential sources of heterogeneity across studies, enhancing the reliability and applicability of our findings.

Nevertheless, our review has limitations that warrant discussion. Most included studies were retrospective, introducing potential biases and limiting real-world applicability. Some used selective sampling (e.g., confirmed fractures vs. clear controls), which may inflate accuracy estimates. Many studies had small sample sizes, and some subgroup analyses involved few events, leading to wide confidence intervals. Although analyses were conducted per radiograph, paediatric examinations often include multiple projections per encounter, and residual within-patient clustering may have led to underestimation of uncertainty. We also observed substantial heterogeneity across studies due to variations in design, populations, and statistical measures, which, despite using random-effects modelling and subgroup analysis, remains partly unexplained. Additionally, our analysis was conducted on a per-radiograph basis rather than per-patient, which, while methodologically appropriate under PRISMA-DTA guidance, limits subject-level clinical inference, and most AI models lacked external validation, raising concerns about generalisability. Because our inclusion criteria required a human expert reference standard, head-to-head comparisons directly evaluating the superiority of AI models over radiologists could not be assessed. Publication bias may have skewed results toward more favourable outcomes. We acknowledge that some commercially deployed AI systems report paediatric fracture performance in regulatory or health technology assessment documents; however, these sources rarely provide peer-reviewed, extractable diagnostic accuracy data suitable for PRISMA-DTA–compliant meta-analysis and were therefore outside the predefined scope of this review. Finally, our focus on appendicular fractures and English-only studies may limit the applicability of findings to other anatomical regions and non-English literature.

Future directions: To further the development and safe implementation of AI in paediatric fracture diagnosis, several avenues for future research emerge. First, prospective validation studies are needed to test AI models in real-world clinical settings, such as the ED, assessing their impact on diagnostic accuracy, workflow efficiency, and patient outcomes. Such studies will provide high-quality evidence on the clinical utility of AI tools beyond the controlled research environment. Second, future research should be multicentre and include diverse populations to improve generalisability across institutions and patient subgroups. Third, integration into clinical workflows should be optimised—AI could serve as a triage tool or interactive second reader to enhance decision-making. Additionally, studies should evaluate cost-effectiveness, workflow efficiency, and unintended consequences such as over-reliance or alert fatigue. Finally, AI tools must be continuously refined using new data, but within frameworks that ensure ongoing external validation, monitoring, and audit to maintain safety and utility.

## Conclusion

AI models, particularly deep learning architectures, demonstrate high diagnostic accuracy for detecting paediatric appendicular fractures on radiographs, approaching expert-level performance and improving the diagnostic abilities of junior clinicians. Despite promising results, most evidence comes from retrospective and internally validated studies, raising concerns about generalisability. Future research should prioritise prospective multicentre validation, workflow integration, and assessment of clinical impact before widespread clinical adoption. AI has the potential to become a valuable adjunct in paediatric fracture diagnosis, enhancing detection accuracy and optimising care pathways, but its implementation must be guided by robust evidence, ethical oversight, and clear clinical protocols.

## Abbreviations

AUC: Area under the curve
AI: Artificial intelligence
CNNs: Convolutional neural networks
DOR: Diagnostic odds ratio
ED: Emergency departments
FN: False negative
FP: False positive
HSROC: Hierarchical summary receiver operating characteristic
LR⁻: Negative likelihood ratio
LR^+^: Positive likelihood ratio
TN: True negative
TP: True positive

## References

[1] Farrell C, Hannon M, Monuteaux MC, Mannix R, Lee LK (2022) Pediatric fracture epidemiology and US emergency department resource utilization. Pediatr Emerg Care 38:e1342–e1347 35686967 10.1097/PEC.0000000000002752

[2] Truong WH, Howard AW, Georgiadis AG (2020) Displaced distal radius fractures in children: to reduce or not to reduce? To pin or not to pin? J Pediatr Orthop Soc North Am 2:77

[3] Jones IE, Williams SM, Dow N, Goulding A (2002) How many children remain fracture-free during growth? A longitudinal study of children and adolescents participating in the Dunedin Multidisciplinary Health and Development Study. Osteoporos Int 13:990–995 12459942 10.1007/s001980200137

[4] Cooper C, Dennison EM, Leufkens HG, Bishop N, van Staa TP (2004) Epidemiology of childhood fractures in Britain: a study using the general practice research database. J Bone Min Res 19:1976–1981 10.1359/JBMR.040902 15537440

[5] George MP, Bixby S (2019) Frequently missed fractures in pediatric trauma: a pictorial review of plain film radiography. Radiol Clin North Am 57:843–855 31076036 10.1016/j.rcl.2019.02.009

[6] Eakins C, Ellis WD, Pruthi S et al (2012) Second opinion interpretations by specialty radiologists at a pediatric hospital: rate of disagreement and clinical implications. AJR Am J Roentgenol 199:916–920 22997387 10.2214/AJR.11.7662

[7] Taves J, Skitch S, Valani R (2018) Determining the clinical significance of errors in pediatric radiograph interpretation between emergency physicians and radiologists. CJEM 20:420–424 28625198 10.1017/cem.2017.34

[8] Klein EJ, Koenig M, Diekema DS, Winters W (1999) Discordant radiograph interpretation between emergency physicians and radiologists in a pediatric emergency department. Pediatr Emerg Care 15:245–248 10460076

[9] Al-Sani F, Prasad S, Panwar J et al (2020) Adverse events from emergency physician pediatric extremity radiograph interpretations: a prospective cohort study. Acad Emerg Med 27:128–138 31702075 10.1111/acem.13884

[10] Kavak N, Kavak RP, Güngörer B et al (2024) Detecting pediatric appendicular fractures using artificial intelligence. Rev Assoc Méd Bras 70:e20240523 39230068 10.1590/1806-9282.20240523 PMC11371126

[11] Yang S, Yin B, Cao W, Feng C, Fan G, He S (2020) Diagnostic accuracy of deep learning in orthopaedic fractures: a systematic review and meta-analysis. Clin Radiol 75:713.e717–713.e728 10.1016/j.crad.2020.05.021 32591230

[12] Langerhuizen DW, Janssen SJ, Mallee WH et al (2019) What are the applications and limitations of artificial intelligence for fracture detection and classification in orthopaedic trauma imaging? A systematic review. Clin Orthop Relat Res® 477:2482–2491 31283727 10.1097/CORR.0000000000000848 PMC6903838

[13] Choi JW, Cho YJ, Lee S et al (2020) Using a dual-input convolutional neural network for automated detection of pediatric supracondylar fracture on conventional radiography. Invest Radiol 55:101–110 31725064 10.1097/RLI.0000000000000615

[14] Gan K, Xu D, Lin Y et al (2019) Artificial intelligence detection of distal radius fractures: a comparison between the convolutional neural network and professional assessments. Acta Orthop 90:394–400 30942136 10.1080/17453674.2019.1600125 PMC6718190

[15] Hayashi D, Kompel AJ, Ventre J et al (2022) Automated detection of acute appendicular skeletal fractures in pediatric patients using deep learning. Skelet Radiol 51:2129–2139 10.1007/s00256-022-04070-0 35522332

[16] Shelmerdine SC, White RD, Liu H, Arthurs OJ, Sebire NJ (2022) Artificial intelligence for radiological paediatric fracture assessment: a systematic review. Insights Imaging 13:94 35657439 10.1186/s13244-022-01234-3 PMC9166920

[17] McInnes MDF, Moher D, Thombs BD et al (2018) Preferred reporting items for a systematic review and meta-analysis of diagnostic test accuracy studies: the PRISMA-DTA statement. JAMA 319:388–396 29362800 10.1001/jama.2017.19163

[18] Ufuk F, Agladioglu K, Karabulut N (2016) CT evaluation of medial clavicular epiphysis as a method of bone age determination in adolescents and young adults. Diagn Interv Radiol 22:241–246 27015321 10.5152/dir.2016.15355 PMC4859740

[19] Hasan SU, Siddiqui MAR (2023) Diagnostic accuracy of smartphone-based artificial intelligence systems for detecting diabetic retinopathy: a systematic review and meta-analysis. Diabetes Res Clin Pract 205:110943 37805002 10.1016/j.diabres.2023.110943

[20] Pervez A, Hasan SU, Hamza M et al (2024) Diagnostic accuracy of tests for tuberculous pericarditis: a network meta-analysis. Indian J Tuberc 71:185–194 38589123 10.1016/j.ijtb.2023.05.013

[21] Whiting PF, Rutjes AW, Westwood ME et al (2011) QUADAS-2: a revised tool for the quality assessment of diagnostic accuracy studies. Ann Intern Med 155:529–536 22007046 10.7326/0003-4819-155-8-201110180-00009

[22] Chen P, Liu S, Lu W, Lu F, Ding B (2024) WCAY object detection of fractures for X-ray images of multiple sites. Sci Rep 14:26702 39496710 10.1038/s41598-024-77878-6 PMC11535499

[23] England JR, Gross JS, White EA, Patel DB, England JT, Cheng PM (2018) Detection of traumatic pediatric elbow joint effusion using a deep convolutional neural network. AJR Am J Roentgenol 211:1361–1368 30300006 10.2214/AJR.18.19974

[24] Ju R-Y, Cai W (2023) Fracture detection in pediatric wrist trauma X-ray images using YOLOv8 algorithm. Sci Rep 13:20077 37973984 10.1038/s41598-023-47460-7 PMC10654405

[25] Malek S, Gunalan R, Kedija S et al (2016) A primary study on application of artificial neural network in classification of pediatric fracture healing time of the lower limb. In: Proceedings of the 10th International Conference on Practical Applications of Computational Biology & Bioinformatics. Springer, Cham, pp 23–30

[26] Zhou Y, Teomete U, Dandin O et al (2016) Computer-aided detection (CADx) for plastic deformation fractures in pediatric forearm. Comput Biol Med 78:120–125 27684324 10.1016/j.compbiomed.2016.09.013

[27] Binh LN, Nhu NT, Vy VPT et al (2024) Multi-class deep learning model for detecting pediatric distal forearm fractures based on the AO/OTA classification. J Imaging Inf Med 37:725–733 10.1007/s10278-024-00968-4 PMC11031555 38308069

[28] Dupuis M, Delbos L, Veil R, Adamsbaum C (2022) External validation of a commercially available deep learning algorithm for fracture detection in children. Diagn Interv Imaging 103:151–159 34810137 10.1016/j.diii.2021.10.007

[29] Franco PN, Maino C, Mariani I et al (2024) Diagnostic performance of an AI algorithm for the detection of appendicular bone fractures in pediatric patients. Eur J Radiol 178:111637 39053306 10.1016/j.ejrad.2024.111637

[30] Gasmi I, Calinghen A, Parienti JJ, Belloy F, Fohlen A, Pelage JP (2023) Comparison of diagnostic performance of a deep learning algorithm, emergency physicians, junior radiologists and senior radiologists in the detection of appendicular fractures in children. Pediatr Radiol 53:1675–1684 36877239 10.1007/s00247-023-05621-w

[31] Rayan JC, Reddy N, Kan JH, Zhang W, Annapragada A (2019) Binomial classification of pediatric elbow fractures using a deep learning multiview approach emulating radiologist decision making. Radiol Artif Intell 1:e180015 33937781 10.1148/ryai.2019180015 PMC8017418

[32] Starosolski ZA, Kan JH, Annapragada A (2020) CNN-based detection of distal tibial fractures in radiographic images in the setting of open growth plates. In: Medical Imaging 2020: Computer-Aided Diagnosis. Proceedings of SPIE, Vol. 11314, pp 855–862

[33] Tsai A, Kleinman PK (2022) Machine learning to identify distal tibial classic metaphyseal lesions of infant abuse: a pilot study. Pediatr Radiol 52:1095–1103 35147714 10.1007/s00247-022-05287-w

[34] Zech JR, Carotenuto G, Igbinoba Z et al (2023) Detecting pediatric wrist fractures using deep-learning-based object detection. Pediatr Radiol 53:1125–1134 36650360 10.1007/s00247-023-05588-8

[35] Zech JR, Jaramillo D, Altosaar J, Popkin CA, Wong TT (2023) Artificial intelligence to identify fractures on pediatric and young adult upper extremity radiographs. Pediatr Radiol 53:2386–2397 37740031 10.1007/s00247-023-05754-y

[36] Kuo RYL, Harrison C, Curran TA et al (2022) Artificial intelligence in fracture detection: a systematic review and meta-analysis. Radiology 304:50–62 35348381 10.1148/radiol.211785 PMC9270679

[37] Urakawa T, Tanaka Y, Goto S, Matsuzawa H, Watanabe K, Endo N (2019) Detecting intertrochanteric hip fractures with orthopedist-level accuracy using a deep convolutional neural network. Skelet Radiol 48:239–244 10.1007/s00256-018-3016-3 29955910

[38] Chung SW, Han SS, Lee JW et al (2018) Automated detection and classification of the proximal humerus fracture by using deep learning algorithm. Acta Orthop 89:468–473 29577791 10.1080/17453674.2018.1453714 PMC6066766

[39] Olczak J, Fahlberg N, Maki A et al (2017) Artificial intelligence for analyzing orthopedic trauma radiographs. Acta Orthop 88:581–586 28681679 10.1080/17453674.2017.1344459 PMC5694800

[40] Ashworth E, Allan E, Pauling C, Laidlow-Singh H, Arthurs OJ, Shelmerdine SC (2025) Artificial intelligence (AI) in radiological paediatric fracture assessment: an updated systematic review. Eur Radiol. 10.1007/s00330-025-11449-9 10.1007/s00330-025-11449-9 40063108

[41] Ximenes GF, Costa ÁL, Leite LL et al (2025) Are artificial intelligence models reliable for clinical application in pediatric fracture detection on radiographs? A systematic review and meta-analysis. Clin Orthop Relat Res. 10.1097/corr.0000000000003660 10.1097/CORR.0000000000003660 40839831

[42] Macaskill P, Gatsonis C, Deeks JJ, Harbord RM, Takwoingi Y. Chapter 10: Analysing and Presenting Results. In: Deeks JJ, Bossuyt PM, Gatsonis C (editors), Cochrane Handbook for Systematic Reviews of Diagnostic Test Accuracy Version 1.0. The Cochrane Collaboration, 2010. Available from: http://srdta.cochrane.org/

[43] Janisch M, Apfaltrer G, Hržić F et al (2022) Pediatric radius torus fractures in x-rays—how computer vision could render lateral projections obsolete. Front Pediatr 10:1005099 36589159 10.3389/fped.2022.1005099 PMC9794847

[44] Altmann-Schneider I, Kellenberger CJ, Pistorius S-M et al (2024) Artificial intelligence-based detection of paediatric appendicular skeletal fractures: performance and limitations for common fracture types and locations. Pediatr Radiol 54:136–145 38099929 10.1007/s00247-023-05822-3 PMC10776701

[45] Dupuis M, Delbos L, Rouquette A, Adamsbaum C, Veil R (2024) External validation of an artificial intelligence solution for the detection of elbow fractures and joint effusions in children. Diagn Interv Imaging 105:104–109 37813759 10.1016/j.diii.2023.09.008

[46] Nguyen T, Maarek R, Hermann A-L et al (2022) Assessment of an artificial intelligence aid for the detection of appendicular skeletal fractures in children and young adults by senior and junior radiologists. Pediatr Radiol 52:2215–2226 36169667 10.1007/s00247-022-05496-3

[47] Yogendra PM, Goh AGW, SY Yee et al (2024) Accuracy of radiologists and radiology residents in detection of paediatric appendicular fractures with and without artificial intelligence. BMJ Health Care Inform 31:e101091 10.1136/bmjhci-2024-101091 PMC11624698 39638562

[48] Ciet P, Eade C, Ho ML et al (2024) The unintended consequences of artificial intelligence in paediatric radiology. Pediatr Radiol 54:585–593 37665368 10.1007/s00247-023-05746-y

[49] Chng SY, Tern MJW, Lee YS et al (2025) Ethical considerations in AI for child health and recommendations for child-centered medical AI. NPJ Digit Med 8:152 40065130 10.1038/s41746-025-01541-1 PMC11893894

[50] Wagner MW, Ertl-Wagner BB (2024) Accuracy of information and references using ChatGPT-3 for retrieval of clinical radiological information. Can Assoc Radiol J 75:69–73 37078489 10.1177/08465371231171125

